# Comparison of Radiographic Reconstruction and Clinical Improvement between Artificial Cervical Disc Replacement and Anterior Cervical Discectomy and Fusion

**DOI:** 10.1155/2022/3353810

**Published:** 2022-01-24

**Authors:** Yuxiang Chen, Yue Li, Yong Hai, Peng Yin, Yuzeng Liu, Jincai Yang, Qingjun Su

**Affiliations:** Department of Orthopaedic, Beijing Chao-Yang Hospital, Capital Medical University, No. 8 Gong Ti Nan Road, Chaoyang, Beijing 100020, China

## Abstract

**Background:**

The surgical management of cervical degenerative disc degeneration (CDDD) has not reached a consensus. Artificial cervical disc replacement (ACDR) has been shown to be efficient in reducing symptoms after CDDD, although the topic remains highly controversial in this field. This study aimed to evaluate the effectiveness of ACDR on the treatment of CDDD on the aspect of radiographic reconstruction and clinical improvement compared with anterior cervical discectomy and fusion (ACDF).

**Methods:**

This was a retrospective comparative study with 47 patients who underwent single-level ACDR and 46 patients who underwent single-level ACDF. The radiographic reconstruction was assessed by the cervical sagittal alignment parameters, consisting of two aspects, distance and angle, such as cervical sagittal vertical axis (cSVA), cervical lordosis (CL), T1 slope (T1s), and intervertebral space height (ISH). The clinical improvement was assessed by patient-related outcomes (PROs), consisting of two aspects, relief of axial neck pain and recovery of cervical dysfunction, measured through the Visual Analogue Scale (VAS), Neck Disability Index (NDI), and Japanese Orthopedic Association (JOA).

**Results:**

Significant variations were achieved on aspects of radiographic reconstruction and clinical improvement after ACDR (*P* < 0.05), which were similar to that of the ACDF group (*P* < 0.05). A significantly larger postoperative range of motion (ROM) was found in patients less than 45 years of age in the ACDR group (*P* < 0.05). In addition, a significantly better postoperative JOA was found in patients with a preoperative ISH less than 4 mm in the ACDF group than that in the ACDR group (*P* < 0.05). Other than that mentioned above, no significant variations in radiographic and clinical outcomes were found between the two groups (*P* > 0.05).

**Conclusions:**

Overall, this study showed that a similar capability in terms of radiographic reconstruction and clinical improvement was found between the two methods. Specific concerns should be analyzed while choosing between an ACDR and an ACDF. It should be pointed out that, based on our experience, if the patient is younger, ACDR is recommended; for patients with preoperative ISH less than 4 mm, ACDF is more recommended.

## 1. Background

Myelopathy, radiculopathy, or both can be caused by cervical degenerative disc disease (CDDD) which can induce severe axial neck pain [1]. It has been decades since anterior cervical discectomy and fusion (ACDF) was first introduced and has been regarded as the gold standard procedure to treat CDDD [2–5]. Despite its proven success, ACDF may interfere with cervical sagittal alignment and lead to adjacent segment disease (ASD) [6, 7] due to a decreased range of motion (ROM) at the index level and an increased ROM at the adjacent segment [8–11].

With its improved preservation of the spinal kinematic, artificial cervical disc replacement (ACDR) has been offered as an alternative technique [12, 13], which is supported by both clinical and biomechanical research [14, 15]. In earlier research, ACDF was used as a control group to explore the efficacy of ACDR. However, even on this premise, the optimum treatment remains in dispute [16–24].

The cervical sagittal alignment has a crucial role in transferring axial loads, maintaining horizontal gaze and global spinal balance [25, 26]. It has been shown that dysfunction of the neck and severe axial neck pain is related to abnormal cervical sagittal alignment [27, 28]. To quantify the cervical sagittal alignment, the cervical sagittal vertical axis (cSVA), cervical lordosis (CL), T1 slope (T1s), intervertebral space height (ISH), etc. were assessed in previous research [6, 7, 25, 26]. However, the cervical sagittal alignment has been fiercely disputed, with some studies showing it to be closely associated with patient-reported outcomes (PROs) and others having ambiguous views on the matter [26].

It was, therefore, decided to compare the effect on the aspects of radiographic reconstruction and clinical improvement for patients with CDDD between ACDR and ACDF. The results were expected to be used to provide suitable guidance to surgeons and to assist the prescription for patients.

## 2. Methods

### 2.1. Study Design

The patients diagnosed with CDDD who underwent ACDR or ACDF performed by a single surgeon team from February 2016 to February 2019 in our center were screened for enrollment. All the medical records, radiographic examinations, and clinically functional outcomes were reviewed retrospectively. The present study was approved by the institutional review board of Beijing Chao-Yang Hospital, and written consent was obtained from all the patients preoperatively.

### 2.2. Inclusion and Exclusion Criteria

Inclusion criteria: (1) received either single-level ACDR or ACDF treatment in which the follow-up period was at least twelve months; (2) age: 18–65 years; (3) index level occurred between C3 and C7; and (4) conservative therapy with ineffectiveness.

Exclusion criteria: (1) traumatic injury; (2) tumor; (3) ossification of the posterior longitudinal ligament (OPLL); (4) autoimmune or metabolic bone disease such as ankylosing spondylitis and rheumatoid arthritis; (5) osteoporosis (T-score ≤ −2.5); (6) kyphotic deformity; and (7) prior surgery.

### 2.3. Surgical Indication and Procedure

The indications of ACDR were anterior cervical decompression was required for radiculopathy and/or myelopathy; the contraindications of ACDR were malalignment of the cervical spine, severe kyphosis, obvious instability, advanced age, and disc space collapse. Patients with the contraindication mentioned above underwent ACDF, whereas those without underwent ACDR [29, 30]. Also, the surgical procedure and details of ACDR (Mobi-C: Zimmer Biomet) and ACDF (Cage: Medtronic) in this study by the same surgeon team were in accordance with previous studies [14, 30–33].

### 2.4. Clinical Measurement

The clinical improvement was assessed by patient-related outcomes (PROs), consisting of two aspects: relief of axial neck pain evaluated by the Visual Analogue Scale (VAS) and recovery of cervical dysfunction assessed via the score of Neck Disability Index (NDI) and the Japanese Orthopedic Association (JOA) score. For the VAS and NDI, a decrease represents an improvement, whereas for the JOA, an increase indicates an improvement.

### 2.5. Radiographic Measurement

**Figure 1:**
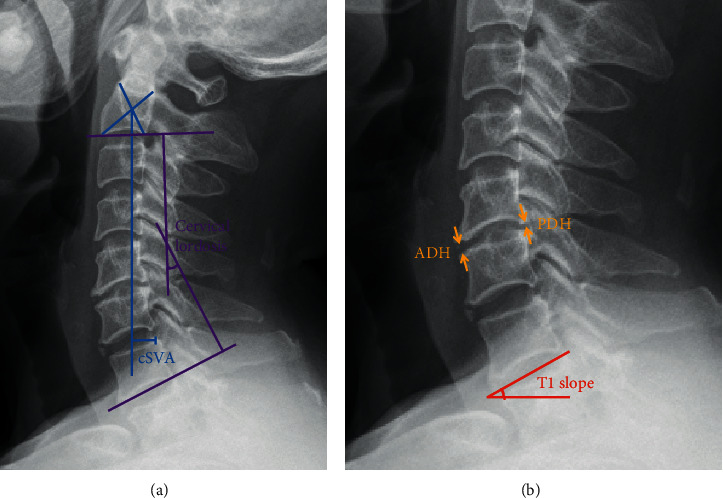
Radiographic measurement of the cervical sagittal alignment parameters. (a) cSVA: the distance between the plumb line from the center of C2 and the superior posterior corner of C7; CL: the angle between C2 (the inferior endplate) and C7 (the inferior endplate); (b) T1s: the angle between T1 (the superior endplate) and a horizontal line; ISH: average of anterior disc height (ADH) and posterior disc height (PDH). Abbreviations: cSVA, cervical sagittal vertical axis; CL, cervical lordosis; T1s, T1 slope; ISH, intervertebral space height; ADH, anterior disc height; and PDH, posterior disc height.

The radiographic reconstruction was assessed by the cervical sagittal alignment parameters, consisting of two aspects, distance and angle, such as cervical sagittal vertical axis (cSVA), cervical lordosis (CL), T1 slope (T1s), and intervertebral space height (ISH) (Figure 1). Preoperative and postoperative radiographs were obtained, as well as at the follow-up.

### 2.6. Statistical Analysis

Mean and standard deviation was used to represent results in the study. Student's *t*-test and ANOVA were utilized in this study. It was deemed statistically significant if the two-tailed *P* < 0.05. All statistical analyses were performed using GraphPad Prism 8.

## 3. Results

### 3.1. Patients' Baseline Characteristics

Demographic information and surgical data are reported in Table 1.

Patients in this study consisted of 93 individuals with complete baseline and follow-up data. The mean patient age was 48.73 ± 11.31 years, mean body mass index (BMI) was 25.30 ± 3.903 kg/m^2^, mean follow-up was 47.40 months (from 30 to 66 months) (Table 2), and 39% of patients were female. The series in the ACDR group was younger than that in the ACDF group (*P* < 0.05). A total of 62 patients had an index level of C5 to C6, who are most likely to develop CDDD based on previous research.

### 3.2. Clinical Improvement Outcomes

After surgery, both groups of patients received significant relief of neck pain and improvement of dysfunction of the cervical spine, and the results are summarized in Table 3.

The preoperative VAS was 7.617 ± 1.114 and 7.674 ± 1.055 in the ACDR and ACDF groups. Both of the two groups achieved significant pain relief to 1.511 ± 0.5053 and 1.435 ± 0.5437, respectively (*P* < 0.05). In addition, the preoperative NDI was 80.68 ± 5.129 and 79.30 ± 5.219 in the ACDR and ACDF groups, respectively. The value of NDI in the ACDR and ACDF groups decreased considerably at the follow-up to 26.26 ± 17.210 and 27.70 ± 14.250, respectively (*P* < 0.05). Furthermore, a similar clinical improvement result was found in the value of the JOA score. The JOA in the ACDR group improved from 6.120 ± 1.156 to 11.850 ± 1.609, and that improved from 6.554 ± 1.671 to 12.460 ± 1.807 in the ACDF group (*P* < 0.05). However, no significant differences were found between the two groups at the time point of the follow-up (*P* > 0.05).

### 3.3. Radiographic Reconstruction Outcomes

Radiographic reconstruction improved similarly in both two groups and is reported in Tables 4 and 5. Cervical alignment parameters such as cSVA, CL, T1s, and ISH were significantly improved (*P* < 0.05). The follow-up also found that they were significantly improved than those before surgery (*P* < 0.05). Although a significant difference of cervical alignment parameters (cSVA, CL, T1s, and ISH) was found in each group at each time point, there were no significant differences between the two groups (*P* > 0.05).

Referring to motion preservation, ACDR was designed for the maintenance of ROM at the original. With no surprises, a significant improvement was found among all the time points (preoperative, postoperative, and follow-up) in the ACDR group (*P* < 0.05). However, the statistical improvement was only found from preoperative to postoperative in the ACDF group, no significant differences were found neither from postoperative to follow-up nor from preoperative to follow-up (*P* > 0.05). Furthermore, a statistical difference at the time point of postoperative and follow-up between the two groups was found, indicating that there was an advantage in ACDR compared with ACDF on the aspect of ROM maintenance, which was in line with our expectations.

## 4. Discussion

The cervical sagittal alignment has gained great attention as an important factor to determine axial neck pain and dysfunction of the cervical spine. Modifications to the cervical sagittal alignment might increase tiredness and neck discomfort [34, 35]. For this reason, it is important to maintain or reconstruct cervical sagittal alignment after spine surgery. This is also the reason why the cervical sagittal alignment has been used to evaluate the reconstruction of the cervical spine.

Except for the intrinsic difference between the abovementioned two surgeries, the present study estimated the effect on cervical sagittal alignment and PROs of the ACDR versus ACDF and discovered that both the two methods could do well for the treatment of CDDD.

Regarding the aspect of the patient-related outcomes, the value of VAS, NDI, and JOA scores showed statistically significant improvement in both ACDR and ACDF groups which were in accordance with the findings of diverse kinds of ACDR studies. But the two groups were evenly matched in terms of this aspect in this study [36–41].

When it comes to determining cervical sagittal alignment, the value of cSVA is often used as a key metric. With C7 as the foundation of support, cSVA stands for cervical spine offset. In addition, postoperative cSVA >40 mm has been observed to be associated with poor PROs [42]. Furthermore, Iyer et al. believed that cSVA is an independent predictor of preoperative NDI [28]. Similarly, the present study found that preoperative ISH is associated with postoperative JOA.

It has to be said that the disadvantage of ACDR, in the beginning, is the inability to restore the sagittal curvature of the cervical spine. Kim et al. conducted retrospective research on the utilization of the ACDR prosthesis and reported that only 36% of the patients retained CL 33 months after the operation [43]. With technological advancement, studies have shown that the prosthesis can maintain the sagittal curvature of the cervical spine compared with that preoperative, through strict criteria and improved operation, but it can only maintain but not reconstruct the cervical alignment. In the ACDR group in this study, the value of CL was improved from 15.97 ± 11.55 preoperatively to 20.58 ± 12.31 postoperatively and 19.00 ± 11.62 at follow-up with a significant difference compared with baseline. The result of this study showed that CL could be reconstructed well through ACDR. The authors analyzed the reasons and considered that it may be due to the absolute removal of osteophyte, repairment of the endplate bed, and suitable choice of the prosthesis.

According to a prospective study by Lee et al. [44], the greater value of CL was associated with a greater value of T1s, which had a key role to preserve the physiological neck tilting and horizontal gaze and determine the sagittal balance of the cervical spine. The forward inclination of T1 can lead to the forward movement of the center of gravity of the cervical spine. In addition, the stability of the posterior cervical muscle makes the cervical lordosis increase and the head move backward, to make the balance center of gravity forward. As a result, a greater T1 inclination requires a larger CL to maintain the cervical sagittal alignment balance, representing the relationship between the T1s and global sagittal alignment [45]. In the present study, the value of T1s achieved significant improvement from 20.44 ± 6.057 preoperative to 26.52 ± 4.954 postoperative in the ACDR group and 21.13 ± 5.696 preoperative to 27.72 ± 5.777 postoperative in the ACDF group. Both the two groups had a certain degree of loss, which was 24.79 ± 4.937 and 25.82 ± 5.477 at follow-up, respectively. The changing trend between T1s, cSVA, and CL in this study was consistent with that of previous studies [44–47].

It is also necessary to note that restoring and maintaining the value of ISH is of importance to reconstruct the cervical sagittal alignment. It is closely associated with axial neck pain, adjacent segment degeneration, and neurologic symptoms [48]. Liu et al. conducted a clinical study on the aspect of the association between ISH and ROM to explore the efficacy of ACDR.

No correlation was found between the cervical sagittal alignment parameters (cSVA, CL, T1s, and ISH) and PROs (VAS, NDI, and JOA) in previous studies [49], except that patients with preoperative ISH < 4 mm exhibited increased postoperative ROM, while those with preoperative ISH > 4 mm remained the same [48], which was similar with the study of Basques et al. [50]. However, in this study, no similar results were found in the ACDR group. The authors in this study considered that it might be due to the difference in the prosthesis, Bryan was utilized in theirs while Mobi-C was used in the present study. Additionally, a significantly better postoperative JOA was found in patients with a preoperative ISH < 4 mm in the ACDF group than the ACDR group in this study. These results may interpret that patients with more preoperative loss of ISH may suffer from low quality of life such as intolerable severe axial neck pain and need more thorough decompression during the operation, even including the resection of the posterior longitudinal ligament; as a result, the improvement postoperative may be changed significantly compared with that with less preoperative loss of ISH. For the abovementioned reasons, the authors believed that, in patients with preoperative ISH less than 4 mm, ACDF was more recommended.

In addition to the abovementioned findings, the results of this study also showed a difference in the baseline of age. Reviewing the data, the authors found that the median age of enrolled patients in this study was exactly 45 years. The author analyzed and considered that it may due to the different surgical indications of the ACDR and ACDF in daily clinical practice. Also, this study aimed to compare the radiographic reconstruction and clinical improvement between the two procedures, so the author held that it would not unduly affect the pooled results and conclusions. Additionally, the author divided the enrolled patients into groups of less than 45 years of age and more than 46 years of age to verify whether the daily clinical experience of recommending ACDR for younger patients and ACDF for elderly patients should continue to be followed. The result showed that patients with age less than 45 years received a significantly larger postoperative ROM than patients with age more than 46 years in the ACDR group (*P* < 0.05). However, a similar result was not found in the ACDF group (*P* > 0.05).

There were also limitations in the present study. Only the Mobi-C prosthesis was included in this study, and the results may be modified by diverse kinds of different prostheses, and the sequential studies were still needed and went on. Also, prospective cohorts with various types of prosthesis as well as higher sample sizes might support stronger findings.

## 5. Conclusions

Overall, according to the results mentioned above, both the two methods could do well for the treatment of CDDD, and a similar capability in terms of radiographic reconstruction and clinical improvement was found between the two methods. Specific concerns should be analyzed while choosing between an ACDR and an ACDF. It should be pointed out that, based on our experience, if the patient is younger, ACDR is recommended; for patients with preoperative ISH less than 4 mm, ACDF is more recommended.

## Figures and Tables

**Table 1:** General information of the patients^a^.

Variable	ACDR	ACDF	Total	*P* value
Number of cases	47	46	93	
Gender (female/male)	12/35	24/22	36/57	
Age (years)	45.68 ± 9.255	51.85 ± 12.43	48.73 ± 11.31	0.048
BMI (kg/m^2^)	25.28 ± 3.860	25.33 ± 3.989	25.30 ± 3.903	0.95
Follow-up (months)	48.72 ± 10.39	46.04 ± 10.82	47.40 ± 10.63	0.23
Surgical level				
C3/4	6	6	12	0.62
C4/5	1	2	3	0.50
C5/6	28	32	62	0.26
C6/7	12	4	16	0.052

^a^Values are presented as mean ± standard deviation or number of cases. Abbreviations: ACDR, artificial cervical disc replacement; ACDF, anterior cervical discectomy and fusion.

**Table 2:** Age and postoperative ROM outcome^a^.

Age	ACDR		ACDF	
	*N*	Postoperative ROM	*N*	Postoperative ROM
≤45	24	10.31 ± 3.499	23	7.282 ± 3.189
≥46	23	8.293 ± 2.573	24	6.590 ± 3.673
*P* value	0.0274^b^		0.5210	

^a^Values are presented as mean ± standard deviation. ^b^Significant difference between the age ≤45 group and age ≥46 group. Abbreviations: ROM, range of motion.

**Table 3:** Pain relief and dysfunction improvement^a^.

Group		ACDR	ACDF
VAS	Preoperative	7.617 ± 1.114	7.674 ± 1.055
	Follow-up	1.511 ± 0.5053	1.435 ± 0.5437
	*P* value (preop vs. follow-up)	<0.001^b^	<0.001^b^

NDI	Preoperative	80.68 ± 5.129	79.30 ± 5.219
	Follow-up	26.26 ± 17.210	27.70 ± 14.250
	*P* value (preop vs. follow-up)	<0.001^b^	<0.001^b^

JOA	Preoperative	6.120 ± 1.156	6.554 ± 1.671
	Follow-up	11.850 ± 1.609	12.460 ± 1.807
	*P* value (preop vs. follow-up)	<0.001^b^	<0.001^b^

^a^Values are presented as mean ± standard deviation or number of cases. ^b^Significant difference compared with preoperative values. Abbreviations: VAS, Visual Analogue Scale; NDI, Neck Disability Index; JOA, Japanese Orthopedic Association.

**Table 4:** Cervical sagittal alignment parameters^a^.

ACDR	Preoperative	Postoperative	Follow-up	*P* value (preop vs. postop)	*P* value (preop vs. follow-up)	*P* value (postop vs. follow-up)
cSVA (mm)	10.37 ± 3.816	7.906 ± 3.197	8.558 ± 3.312	0.0058^b^	0.0084^b^	0.0062^b^
CL (°)	15.97 ± 11.55	20.58 ± 12.31	19.00 ± 11.62	0.0014^b^	0.0056^b^	0.0062^b^
T1s (°)	20.44 ± 6.057	26.52 ± 4.954	24.79 ± 4.937	0.0022^b^	0.0055^b^	0.0051^b^
ISH (mm)	4.200 ± 0.7466	6.726 ± 1.071	6.100 ± 0.9377	0.0078^b^	0.0040^b^	0.0044^b^
ROM (°)	4.745 ± 2.253	10.29 ± 3.335	9.151 ± 3.133	0.0037^b^	0.0053^b^	0.0027^b^
ACDF	Preoperative	Postoperative	Follow-up	*P* value (preop vs. postop)	*P* value (preop vs. follow-up)	*P* value (postop vs. follow-up)
cSVA (mm)	12.59 ± 6.798	9.509 ± 6.582	10.40 ± 6.613	0.0085^b^	0.0090^b^	0.0018^b^
CL (°)	15.56 ± 8.636	21.92 ± 9.998	19.96 ± 9.166	0.0046^b^	0.0038^b^	0.0049^b^
T1s (°)	21.13 ± 5.696	27.72 ± 5.777	25.82 ± 5.477	0.0097^b^	0.0036^b^	0.0025^b^
ISH (mm)	4.229 ± 1.177	6.408 ± 1.344	5.770 ± 1.186	0.0056^b^	0.0015^b^	0.0086^b^
ROM (°)	5.467 ± 3.952	7.457 ± 3.797	6.846 ± 3.482	0.0157^b^	0.0793	0.4234
					0.0002^c^	0.0011^c^

^a^Values are presented as mean ± standard deviation. ^b^Significant difference between preoperative, postoperative, or follow-up values. ^c^Significant difference between ACDR and ACDF. Abbreviations: cSVA, cervical sagittal vertical axis; CL, C2-7 lordosis; T1s, T1 slope; ISH, intervertebral space height; ROM, range of motion.

**Table 5:** Preoperative ISH and postoperative JOA outcome^a^.

Preoperative ISH (mm)	ACDR		ACDF		*P* value
	*N*	Postoperative JOA	*N*	Postoperative JOA	
≤4	20	11.88 ± 1.700	21	12.95 ± 1.516	0.0383^b^
>4	27	11.72 ± 1.631	25	12.04 ± 1.952	0.5259

^a^Values are presented as mean ± standard deviation. ^b^Significant difference between ACDR and ACDF. Abbreviations: ISH, intervertebral space height; JOA, Japanese Orthopedic Association.

## Data Availability

The data analyzed during the current study are not publicly available due to the data being confidential; however, they are available from the corresponding author on reasonable request.
